# Parallel evolution of circulating FABP4 and NT-proBNP in heart failure patients

**DOI:** 10.1186/1475-2840-12-72

**Published:** 2013-05-04

**Authors:** Anna Cabré, Pilar Valdovinos, Iolanda Lázaro, Gil Bonet, Alfredo Bardají, Lluís Masana

**Affiliations:** 1 Unitat de Recerca en Lípids i Arteriosclerosi, Hospital Universitari Sant Joan, CIBERDEM, Reus, Spain; 2 IISPV, Universitat Rovira i Virgili, Tarragona, Spain; 3 Servei de Cardiologia, Hospital Universitari de Tarragona Joan XXIII, Tarragona, Spain

**Keywords:** FABP4, Heart failure, NT-proBNP, Obesity, Diabetes

## Abstract

**Background:**

Circulating adipocyte fatty acid-binding protein (FABP4) levels are considered to be a link between obesity, insulin resistance, diabetes, and cardiovascular (CV) diseases. *In vitro*, FABP4 has exhibited cardiodepressant activity by suppressing cardiomyocyte contraction. We have explored the relationship between FABP4 and the N-terminal fragment of pro-B-type natriuretic peptide (NT-proBNP) as a clinical parameter of heart failure (HF).

**Methods:**

We included 179 stable HF patients who were referred to a specialized HF unit, 108 of whom were prospectively followed for up to 6 months. A group of 163 non-HF patients attending a CV risk unit was used as the non-HF control group for the FABP4 comparisons.

**Results:**

In the HF patients, FABP4 and NT-proBNP were assayed, along with a clinical and functional assessment of the heart at baseline and after 6 months of specialized monitoring. The FABP4 levels were higher in the patients with HF than in the non-HF high CV risk control group (*p*<0.001). The FABP4 levels were associated with the NT-proBNP levels in patients with HF (r=0.601, *p*<0.001), and this association was stronger in the diabetic patients. FABP4 was also associated with heart rate and the results of the 6-minute walk test. After the follow-up period, FABP4 decreased in parallel to NT-proBNP and to the clinical parameters of HF.

**Conclusions:**

FABP4 is associated with the clinical manifestations and biomarkers of HF. It exhibits a parallel evolution with the circulating levels of NT-proBNP in HF patients.

## Background

Heart failure (HF) is increasing worldwide, with an overall estimated prevalence of 4% and a prevalence of more than 10% in people older than seventy [1]. Overweight and obese patients have an increased risk of developing HF, and metabolic alterations that are strongly related to adiposity, such as metabolic syndrome (MS) and type-2 diabetes, are also associated with an increased risk of HF [2]. The main cardiac disease associated with these metabolic disorders is coronary artery disease, which can lead to HF. However, in obese, MS and type-2 diabetic patients, HF also appears independently of vascular lesions after years of the development of sub-clinical left systolic dysfunction [3]. Diabetic patients are at a two- to five-fold increased risk of HF [4], whereas the attributed risk of HF due to obesity is 14% in women and 8.8% in men, and the risk of incident HF in adults with a body mass index (BMI) > 30 is 1% per year for men and 0.7% per year for women [3]. However, some studies suggest that obesity per se could play a paradoxical beneficial role in HF [5]. The mechanisms leading to cardiac dysfunction in both obesity and diabetes have been intensively investigated, and although several hypotheses have been raised, the basic etiology remains speculative. Among the molecules released in excess by the adipose tissue of obese, MS and diabetic patients is adipocyte fatty acid-binding protein (FABP4). FABP4 is a small cytoplasmic lipid chaperone that plays an important role in the trafficking of fatty acids in subcellular compartments [6]. In animal models, a FABP4 deficiency has been linked to reduced lipolysis and inflammation, as well as protection from the development of hyperinsulinemia, hyperglycemia, insulin resistance, and atherosclerosis [7-9]. FABP4 is highly expressed in adipose tissue and adipocytes but is also produced in macrophages. FABP4 expression in macrophages plays a specific and independent role in experimental atherosclerosis. In humans, FABP4 has been detected in circulation and seems to be a marker of adiposity and body fat distribution [10]. FABP4 levels are increased in overweight and obese subjects compared with those in lean subjects, and several pathologies have been linked to adipose tissue dysfunction, such as MS, type-2 diabetes, atherogenic dyslipidemia, human immunodeficiency-virus associated lipodystrophy, and polycystic ovary syndrome [10-14]. Serum FABP4 levels have been shown to predict the risk of developing both type-2 diabetes and MS [15,16]. Moreover, serum FABP4 has been associated with the presence and degree of cardiovascular disease [17], with an increased risk for secondary cardiovascular events (particularly cardiovascular death) [18], and also with renal dysfunction in patients with stable angina pectoris [19]. Although the function of serum FABP4 has not yet been elucidated, results from recent publications suggest a systemic effect of FABP4 on peripheral tissues. In diabetic subjects, elevated FABP4 levels in the serum have been associated with endothelial dysfunction [20]. *In vitro* studies have shown that FABP4 contributes to endothelial dysfunction [21] and exhibits a cardiomyocyte depressing action [22]. FABP4 inhibits the cell shortening amplitude and the intracellular systolic peak Ca^2+^ in a dose-dependent manner in isolated rat cardiomyocytes, most likely due to a reduced excitation-contraction gain [22]. However, FABP4 is expressed in endothelial cells and this expression could potentially contribute to the FABP4 levels observed in patients with HF. Therefore, this molecule could be considered a link between obesity, MS, type-2 diabetes, and cardiac insufficiency.

In this study, we have explored the hypothesis that circulating FABP4 levels are associated with the biomarkers of HF, such as the N-terminal fragment of pro-B-type natriuretic peptide (NT-proBNP), and with HF clinical parameters.

## Methods

A case–control and cross-sectional study was performed using the basal data and a prospective evaluation after a 6-month follow-up period.

Between March, 2006 and July, 2010, 179 patients in stable HF condition who were referred to a multidisciplinary outpatient HF program in the *Hospital Universitari de Tarragona Joan XXIII* were included. A total of 41.3% of these patients presented ischemic HF, 20.6% presented idiopathic HF, and 21.2% presented hypertensive HF, whereas 16.7% of the patients presented other etiologies. The inclusion criteria were a confirmed diagnosis of HF based on clinical criteria plus a structural and/or functional heart anomaly according to echocardiography, following the diagnostic criteria for HF proposed by the European Society of Cardiology [23].

The basal FABP4 levels were compared to those of an age-, gender-, and BMI-matched group of 163 non-HF patients attending our cardiovascular risk unit for risk factor management.

The investigation conformed to the principles outlined in the Declaration of Helsinki. Written informed consent was obtained from each included subject, following the protocol approved by the Ethics Committee of our hospitals.

During the first visit, the HF patients underwent a complete physical examination that included a careful evaluation of signs and symptoms of congestive HF, a complete assessment of heart function (see below) and the collection of relevant clinical and demographic information. Blood samples were also collected.

A chest x-ray and electrocardiogram were performed for each patient at the same visit. All of the patients underwent a 2-dimensional Doppler echocardiography examination (GE Vivid-7, GE Healthcare, Horten, Norway). The systolic function was quantified by measuring the left ventricular ejection fraction using the Simpson method, according to the American Society of Echocardiography recommendations [24].

The functional status of each patient was assessed using the New York Heart Association classification [25], and the 6-minute walk test (6MWT) was performed according to the standard protocol with the patients in a clinically stable condition that allowed for ambulation without assistance [26]. At baseline, the level of self-care behavior was evaluated using the extensively tested and validated Minnesota Living with Heart Failure Questionnaire (MLHFQ) [26,27]. Similar studies were performed after a 6-month follow-up period.

Blood tests for the lipid profile, glucose, HbA_1c_, and creatinine measurements were performed at baseline and at the 6-month follow-up using standard techniques. The NT-proBNP and FABP4 levels were determined using commercial EIA and ELISA kits (Biomedica, Vienna, Austria and Bio Vendor Laboratory Medicine Inc., Brno, Czech Republic), respectively. The estimated glomerular filtration rate (eGFR) was calculated using the modification of diet in the renal disease equation [28].

SPSS version 17.0 (SPSS Inc., Chicago, IL) was used to carry out all of the statistical analyses. The normality distribution was assessed using the Kolmogorov-Smirnov test. A log-transformation was performed before the analyses when the variables exhibited a skewed distribution. The baseline data are presented as the mean±SD or the median with interquartile range, as appropriate, for the continuous variables and as frequencies for the categorical variables. For the continuous variables, one-way ANOVA was used to compare between groups. The χ^2^ test was used to compare the categorical variables between groups. Spearman correlation tests were used to analyze the bivariate associations between the changes in FABP4 and the changes in other variables. For further correlation studies that excluded the effect of the mentioned confounding variables on each bivariate association, the following adjustments were made: FABP4 and NT-proBNP levels were adjusted for age, gender, BMI, and eGFR; triglycerides and the 6MWT results were adjusted for the eGFR; left ventricular end-diastolic diameter and left ventricular end-systolic diameter were both adjusted for gender; and left ventricular ejection fraction was adjusted for BMI. Statistical comparisons between the correlation coefficients were performed using a Fisher r to Z testing program. The observed differences of FABP4 and NT-proBNP before and after the follow-up period were analyzed using the paired *t*-test. Changes in the variables were calculated as the 6-month follow-up values minus the baseline values. Partial bivariate correlation tests were used to adjust the bivariate associations of changes in FABP4 and NT-proBNP for the confounding variables. In all of the cases, two-sided *p*<0.05 values were considered significant.

## Results

Table 1 shows the baseline characteristics of the subjects and a comparison of the clinical and the biochemical parameters between the HF and non-HF subjects. The cardiac functional parameters and medication histories of the HF group are shown in Table 2. Tables 3 and 4 presents the correlation between FABP4 and NT-proBNP, the other heart function tests and the metabolic characteristics of the HF patients.

**Table 1:** Baseline characteristics of the study patients

**Variables**	**HF subjects (n=179)**	**Non-HF subjects (n=163)**	p^*^
Age (years)^†^	70 (58–76)	68 (57–75)	0.479
Gender (M/F)	132/47	123/40	0.716
Diabetes (%)	35	51	0.002
Hypertension (%)	57	71	0.010
Obesity (%)	41	44	0.807
Waist circumference (cm)	101.8±12.9	102.9±9.8	0.315
BMI (kg/m^2^)	29.0 (26.5−33.1)	29.3 (27.3−31.8)	0.975
SBP (mmHg)^†^	120 (110–140)	137 (130–149)	< 0.001
DBP (mmHg)^†^	70 (66–80)	85 (78–90)	< 0.001
Glucose (mmol/L)^†^	5.9 (5.2–7.3)	6.2 (5.4–7.9)	0.386
Total-cholesterol (mmol/L)	4.72±1.29	5.03±1.11	0.028
LDL-cholesterol (mmol/L)	2.94±1.00	3.09±0.88	0.222
HDL-cholesterol (mmol/L)	1.11 (0.95–1.31)	1.34 (1.16–1.55)	< 0.001
Triglycerides (mmol/L)^†^	1.35 (0.98–1.71)	1.50 (0.98–2.32)	0.008
FABP4 (μg/L)^†^	29.1 (19.2−55.0)	24.2 (19.3−34.5)	< 0.001

Abbreviations: HF, heart failure; BMI, body mass index; SBP, systolic blood pressure; DBP, diastolic blood pressure; LDL, Low-Density Lipoprotein; HDL, High-Density Lipoprotein; FABP4, adipocyte fatty acid binding protein.

The data are presented as the mean±SD or median (interquartile range), unless otherwise indicated.

^*^ANOVA and *x*^2^ tests were used to compare the mean of quantitative variables or the frequency of qualitative traits between HF and non-HF groups of subjects.

^†^Log-transformed before analysis.

**Table 2:** Cardiac function tests and treatments in the HF subjects

**Variables**	**HF subjects (n=179)**
Heart rate (bpm)	76.2±13.6
QRS complex (ms)	120 (98–160)
QTc interval (ms)	449.0 (414.0–478.5)
PR interval (ms)	172.9±29.2
Normal sinus ECG rhythm (%)	68
6MWT (m)	375 (250–450)
MLHFQ	38.6±24.1
LVEDD (mm)	63.5±11.6
LVESD (mm)	50.5±11.2
AI	46.1±9.8
Left ventricular ejection fraction (%)	32 (25–39)
NT-proBNP (pmol/L)	679.8 (405.5–1177.9)
ACE inhibitor (%)	65
β-blocker (%)	61
Diuretic (%)	78
Statin (%)	37

Abbreviations: HF, heart failure; ECG, electrocardiogram; 6MWT, six-minute walk test; MLHFQ, Minnesota Living with Heart Failure Questionnaire; LVEDD, left ventricular end-diastolic diameter; LVESD, left ventricular end-systolic diameter; AI, aortic insufficiency; NT-proBNP, N-terminal fragment of pro-B-type natriuretic peptide; ACE, angiotensin-converting enzyme.

The data are presented as the mean±SD or median (interquartile range), unless otherwise indicated.

**Table 3:** Correlations of FABP4 with metabolic factors in the HF group

	** Unadjusted**		** Adjusted for age, gender, BMI and the eGFR**	
**Variables**	***r***	***p***	***r***	***p***
Age	0.256	0.001	0.064	0.436
Weight	0.198	0.008	0.583	< 0.001
Waist circumference	0.409	< 0.001	0.663	< 0.001
BMI	0.332	< 0.001	0.771	< 0.001
SBP	0.001	0.994	0.205	0.013
DBP	0.035	0.648	0.156	0.061
Glucose	0.053	0.529	0.205	0.018
HbA_1c_	0.197	0.046	0.198	0.052
Total-cholesterol	−0.088	0.291	−0.013	0.884
LDL-cholesterol	−0.141	0.103	−0.045	0.613
HDL-cholesterol	−0.049	0.571	−0.053	0.552
Triglyceride	0.097	0.265	0.601	< 0.001
Creatinine	0.494	< 0.001	0.600	< 0.001
eGFR	−0.570	< 0.001	−0.601	< 0.001

Abbreviations: BMI, body mass index; SBP: systolic blood pressure; DBP, diastolic blood pressure; HbA_1c_, glycated hemoglobin, LDL, Low-Density Lipoprotein; HDL, High-Density Lipoprotein; eGFR, estimated glomerular filtration rate.

**Table 4:** Correlations of FABP4 with cardiac factors in the HF group

	** Unadjusted**		** Adjusted for age, gender, BMI and the eGFR**	
**Variables**	***r***	***p***	***r***	***p***
Pulse pressure	0.025	0.833	0.261	0.034
Heart rate	0.190	0.013	0.150	0.075
6MWT	−0.364	< 0.001	−0.610	< 0.001
MLHFQ	0.194	0.016	0.123	0.163
LVEDD	−0.114	0.175	−0.023	0.796
LVESD	−0.010	0.915	−0.011	0.915
AI	0.112	0.254	0.153	0.135
Left ventricular ejection fraction	0.109	0.166	0.043	0.615
NT-proBNP	0.291	< 0.001	0.601	< 0.001

Abbreviations: 6MWT, six-minute walk test; MLHFQ, Minnesota Living with Heart Failure Questionnaire; LVEDD, left ventricular end-diastolic diameter; LVESD, left ventricular end-systolic diameter; AI, aortic insufficiency; NT-proBNP, N-terminal fragment of pro-B-type natriuretic peptide.

### FABP4 correlates with NT-proBNP

The FABP4 levels were positively correlated with the NT-proBNP levels (r=0.291, *p*<0.001). After making adjustments for age, gender, BMI, and the eGFR, the correlation became stronger (r=0.601, *p*<0.001) Table 3 and Additional file 1: Figure S1A). Considering the strong positive correlation between FABP4 and NT-proBNP, even after excluding the effects of the confounding variables, the relationship between FABP4 and NT-proBNP was further examined. The correlation between FABP4 and NT-proBNP was higher (*p*=0.05) in the HF diabetic subjects (r=0.689, *p*<0.001; Additional file 1: Figure S1B) compared to the non-diabetic HF subjects (r=0.470, p<0.001), and the difference was even more significant (*p*<0.001) in the non-obese HF subjects (r=0.883, *p*<0.001; Additional file 1: Figure S1C) compared to the obese HF subjects (r=0.605, *p*<0.001).

### FABP4 decrease during follow-up correlates with a NT-proBNP decrease

**Figure 1:**
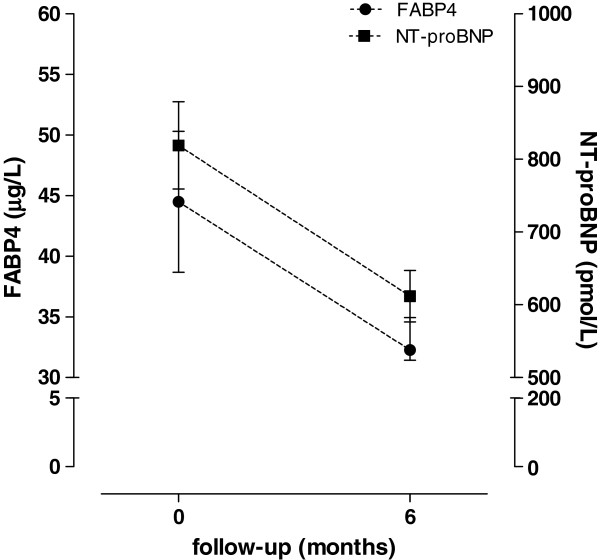
The serum FABP4 (white squares) and NT-proBNP (black squares) concentrations in HF patients at baseline and after 6-months of follow-up.

FABP4 and NT-proBNP were re-evaluated after 6 months of follow-up, at a time when clinical improvements in HF were observed. FABP4 and NT-proBNP plasma concentration reductions occurred in parallel during the follow-up [from 28.0 μg/L (17.4–48.7) to 23.9 μg/L (15.1–39.4) for FABP4, *p*<0.001, and from 641.8 pmol/L (385.4–1073.7) to 540.2 pmol/L (321.4–774.7) for NT-proBNP, *p*<0.001] (Figure 1). The decreases in FABP4 were positively correlated with decreases in NT-proBNP (r=0.307, *p*=0.002) and remained significant after adjusting for age, gender, the eGFR, and BMI at baseline (r=0.306, *p*=0.005).

### Basal FABP4 is higher in HF than in non-HF patients

**Figure 2:**
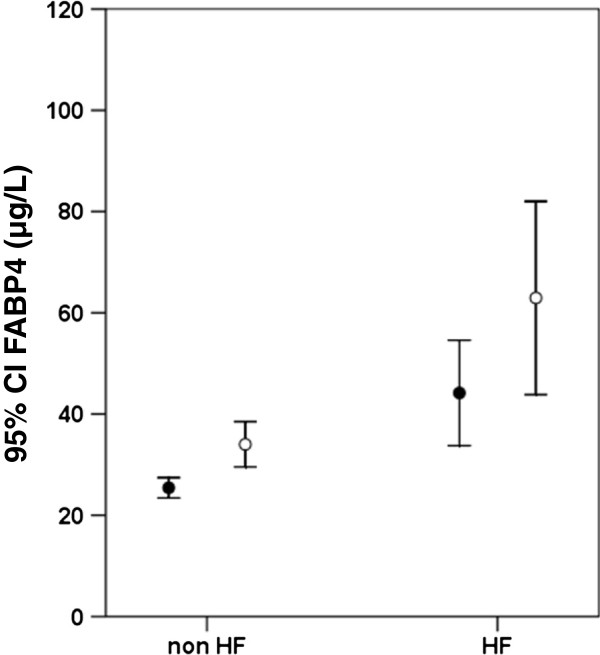
**Comparison of the serum FABP4 levels in non-HF and HF patients, according to gender.** Black circles represent the men, and white circles represent the women.

The subjects with HF had higher FABP4 levels compared to those without HF [29.1 μg/L (19.2–55.0) versus 24.2 μg/L (19.3–34.5), *p*<0.001], despite the fact that there were more subjects with diabetes and atherogenic dyslipidemia in the non-HF group Table 1. The difference between FABP4 levels in the HF and non-HF groups remained significant even after the adjusting for age, gender, BMI, and the presence of diabetes and atherogenic dyslipidemia (*p*<0.001). As shown in Figure 2, the FABP4 levels were significantly higher in women than in men, for both HF subjects [43.6 μg/L (25.2–91.8) versus 27.6 μg/L (17.3–47.7), respectively, *p*=0.001] and non-HF subjects [31.6 μg/L (23.1–41.6) versus 22.5 μg/L (16.2–32.2), respectively, *p*<0.001].

### FABP4 is associated with a clinical assessment of HF

Table 3 presents the relationships between FABP4 and cardiac factors. The FABP4 levels showed significant positive correlations with heart rate (r=0.190, *p*=0.013) and the MLHFQ results (r=0.194, *p*=0.016), as well as a negative correlation with the 6MWT results (r=−0.364, *p*<0.001) Table 4. After adjusting for age, gender, BMI, and the eGFR, the FABP4 levels remained significantly associated with the 6MWT results (r=−0.610, *p*<0.001). Neither FABP4 nor NT-proBNP showed an association with the ejection fraction, as estimated by the Simpson method (r=0.043, *p*=0.615 and r=−0.092, *p*=0.330; respectively).

**Figure 3:**
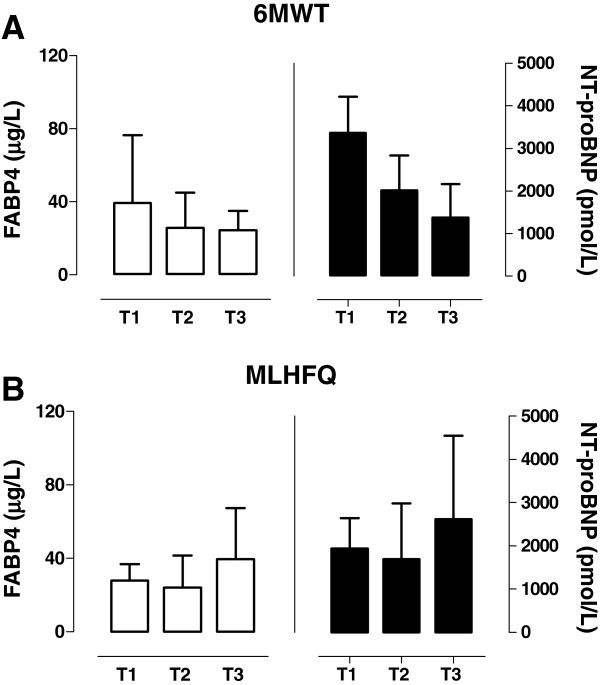
**FABP4 and NT-proBNP relationship with functional status and self-care behavior of HF patients.** The serum FABP4 (white bars) and NT-proBNP (black bars) concentrations in HF patients, stratified by tertiles of the 6MWT results (**A**) and tertiles of the MLHFQ results (**B**).

We further performed a series of analyses to evaluate the association of the FABP4 plasma levels with both the 6MWT results and the MLHFQ results, compared to the NT-proBNP levels. In both comparisons, FABP4 showed a similar tendency to that of NT-proBNP (Figure 3). When all of the HF subjects were divided into 3 groups according to the tertiles of 6MWT results, the FABP4 levels were higher in the HF subjects who presented lower results in the 6MWT results. Considering all of the groups, there was a negative association between FABP4 and the tertiles of 6MWT results [42.2 μg/L (25.9–81.9) versus 26.6 μg/L (15.7–45.9) versus 24.4 μg/L (16.8–35.1), respectively, *p*<0.001] (Figure 3A). Making an adjustment for the covariates did not significantly change the association. Moreover, the FABP4 levels were higher in the group with the highest MLHFQ results, and the overall tendency was toward an increase in FABP4 levels across the tertiles of MLHFQ results [27.9 μg/L (20.3–37.4) versus 24.0 μg/L (16.8–42.4) versus 43.0 μg/L (19.9–75.5), respectively, *p*=0.018] (Figure 3B). However, in this case, making an adjustment for the covariates appreciably changed the significance of the association.

These results suggested that the FABP4 levels were higher in the subjects with a poor quality of life, estimated by both the 6MWT results and the MLHFQ results, and that the FABP4 levels showed a similar pattern to that observed for the NT-proBNP levels.

### FABP4 is associated with obesity and diabetes in HF patients

In the HF subjects, the FABP4 levels were positively associated with age, weight, waist circumference, BMI, HbA_1c_, and serum creatinine levels (all *p*<0.01), as well as inversely associated with the eGFR (*p*<0.001). After adjusting for age, gender, BMI, and the eGFR, the FABP4 levels showed positive correlations with the systolic blood pressure, glucose, and triglycerides (all *p*<0.05), and the relationship between FABP4 and the anthropometry parameters become stronger (r=0.583, *p*<0.001 versus r=0.198, *p*=0.008 for weight, and r=0.663 *p*<0.001 versus r=0.409, *p*<0.001 for waist circumference) Table 3. The obese HF subjects had higher FABP4 levels compared to the non-obese HF subjects [35.3 μg/L (22.3–62.3) versus 25.0 μg/L (16.6–42.5), respectively, *p*=0.004], even after adjusting for age, gender, and the eGFR (*p*<0.001).

The HF subjects with diabetes showed higher FABP4 levels compared to the non-diabetic HF subjects [33.0 μg/L (20.2–76.0) versus 27.8 μg/L (17.2–44.2), respectively, *p*=0.019], even after adjusting for age, gender, BMI and the eGFR (*p*=0.002).

## Discussion

Based on data from basic experiments indicating that *in vitro* FABP4 induces a decrease in the contraction capacity of myocardial cells [22], we addressed the hypothesis that FABP4 is associated with HF. To test the hypothesis, we chose the NT-proBNP sera concentrations as our objective marker of HF. Our results demonstrate a strong relationship between FABP4 and the HF state, as evidenced by the NT-proBNP values. In other words, FABP4 was directly correlated with NT-proBNP in the HF patients. A recent study reported that FABP4 was directly associated with NT-proBNP in Asian coronary artery disease patients [29], and during the review process of this study, the same group showed similar results to ours in HF patients [30]. Moreover, Djoussé et al. reported that the baseline FABP4 levels were associated with the risk of HF in a prospective study but not with HF with or without left ventricular systolic dysfunction [31]. These results are similar to ours (no correlation between FABP4 and the ejection fraction was observed), suggesting that circumstances other than myocardial function determine the association between FABP4 and HF markers. NT-proBNP is a useful biomarker to diagnose and manage HF. It is a helpful tool to establish a HF diagnosis in emergency departments, and the plasma concentration of this molecule is a marker of HF functional status. NT-proBNP has also been proposed as an indicator for follow-up therapy and prognosis [32,33]. The production of NT-proBNP is stimulated by heart volume overload secondary to systolic dysfunction. Therefore, our results, which demonstrate a parallel association between FABP4 and NT-proBNP, support the role of FABP4 as a HF biomarker. Additionally, FABP4 was correlated to other HF clinical markers, mainly heart rate, the 6MWT results, and MLHFQ results, although associations with the left ventricular ejection fraction and telediastolic volume were not observed. The FABP4 levels showed a paralleled association with the NT-proBNP levels during treatment and follow-up, suggesting that an improvement in the HF status was associated with a reduction in both NT-proBNP and FABP4 concentrations, although the underlining mechanism remains unclear. A decrease of more than 30% in the NT-proBNP levels has been associated with a better prognosis for HF [34]. In our follow-up study, the HF patients who showed greater than 30% reductions in the NT-proBNP levels also showed higher decreases in the FABP4 levels. Although a direct effect of treatment cannot be excluded, the drugs affecting the FABP4 levels seem to be restricted to statins and those influencing PPARγ activation, none of which are the main therapeutic interventions for HF treatment. This parallelism between a decrease in NT-proBNP, HF clinical improvement, and a decrease in FABP4 suggests a secondary role of FABP4 concentrations in HF. Therefore, FABP4 may be considered to be a biomarker rather than an etiological agent. The FABP4 levels were higher in the HF patients than in the non-HF group, despite the increased number of subjects who were diabetic and hyperlipidemic in the control group, conditions associated with higher FABP4 concentrations. This observation suggests that HF itself could determine elevations in FABP4 concentrations by currently unknown mechanisms.

The strong association between NT-proBNP and FABP4 was even higher when only the diabetic patients were considered. Because type-2 diabetes is associated with high FABP4 values, a possible link between type-2 diabetes and an increased risk of developing HF cannot be completely excluded. Surprisingly, when obese and non-obese patients were independently analyzed, the association between FABP4 and NT-proBNP was higher in the non-obese group. This association between obesity and HF is paradoxical. It has been reported that obese patients have a reduced risk for HF, and the overall prognosis of HF in obese patients is better. In a recent study, it was observed that non-obese type-2 diabetic patients had a worse prognosis when diagnosed with HF [5]. Our results support this observation.

A strong association between NT-proBNP and FABP4 with the creatinine levels and the eGFR was also detected. The worse the renal function, the higher the biomarker concentrations. We have previously reported that FABP4 concentrations are modified by renal function, which is of special interest in diabetic patients [35]. We could consider that renal function impairment is associated with HF, which would then affect the FABP4 levels; however, when these concentrations were adjusted to take the renal function parameters into account, the associations remained significant. Therefore, although we cannot exclude an impact of renal function on the FABP4 levels and on its association with NT-proBNP in HF patients; our data suggest that this association was independent of renal impairment.

Our work has several limiting factors. The first is the relatively small sample size; however, the strength of the results remains valuable. Second, this is an observational study; therefore, no causal links between FABP4 and HF can be extrapolated.

## Conclusion

The FABP4 circulating levels are strongly associated and exhibit a parallel evolution to the NT-proBNP values in HF patients. The exact mechanism underlying this association is not known. FABP4 could be considered to be a new biomarker for HF, particularly in the context of metabolic disturbances.

## Competing interests

The authors declare that they have no competing interest.

## Authors’ contributions

Design of the study: AC, AB, LM, PV. Acquisition of funding: LM, AB. Management of patients and acquisition of data: PV, GB, AB. Analysis of data: AC, IL, LM, AB, PV. Draft of the manuscript: LM, AC, PV, AB. All of the authors read and approved the final manuscript.

## Supplementary Material

Additional file 1: Figure S1 Association of serum FABP4 levels with NT-proBNP in all HF patients studied (**A**), in HF patients with type 2 diabetes (**B**) and in non-obese HF patients (**C**). Click here for file
