# Supplementary figures and images for: Parallel evolution of circulating FABP4 and NT-proBNP in heart failure patients
Source: Cardiovasc Diabetol. 2013 May 4;12:72. doi: 10.1186/1475-2840-12-72 (PMC3653725; doi:10.1186/1475-2840-12-72)

Figure 1

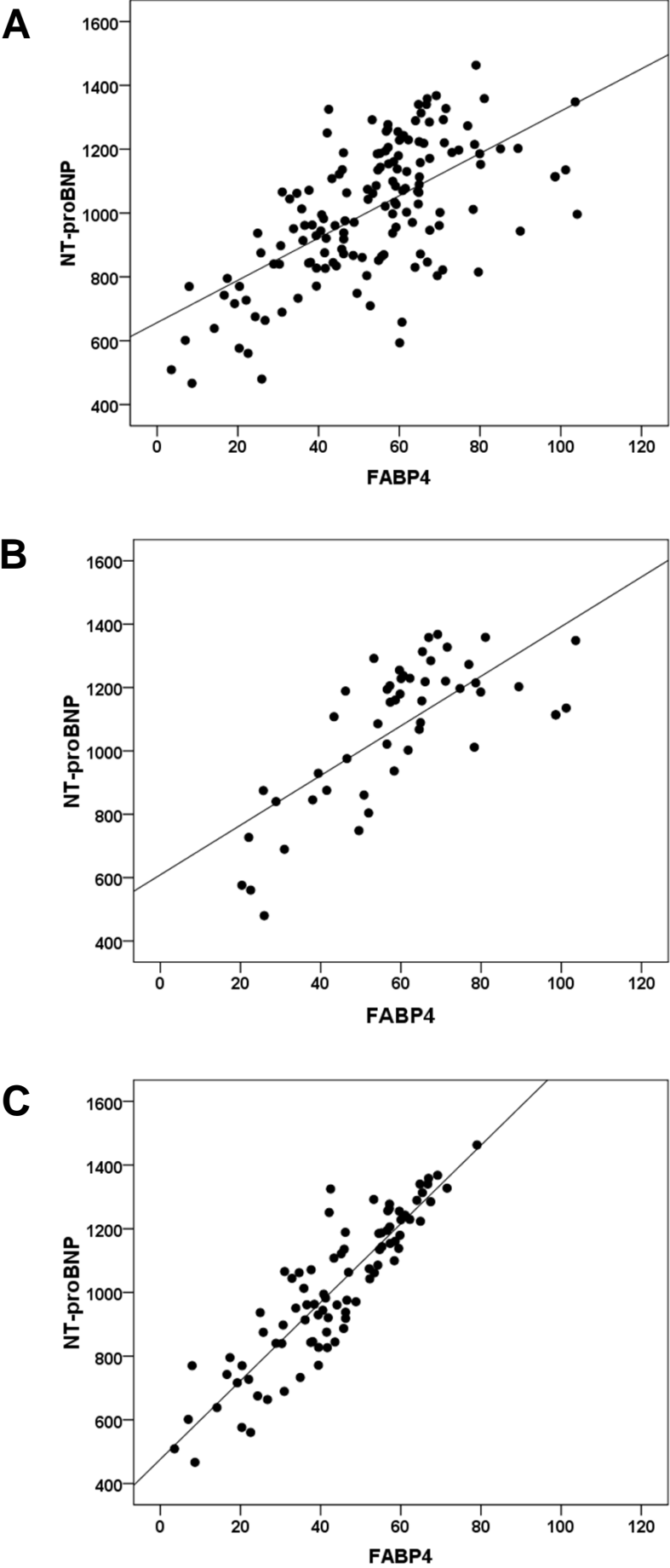

Supplement: Additional file 1: Figure S1 — Association of serum FABP4 levels with NT-proBNP in all HF patients studied (A), in HF patients with type 2 diabetes (B) and in non-obese HF patients (C). [file 1475-2840-12-72-S1.pdf]
